# Ferritin: A Biomarker Requiring Caution in Clinical Decision

**DOI:** 10.3390/diagnostics14040386

**Published:** 2024-02-10

**Authors:** Baptiste Lemaire, Miguel A. Frias, Olivier Golaz, Jean-Luc Magnin, Véronique Viette, Nicolas Vuilleumier, Sophie Waldvogel Abramowski

**Affiliations:** 1 Diagnostic Department, Geneva University Hospitals, 1205 Geneva, Switzerland; 2 Medicine Department, Geneva University Hospitals, 1205 Geneva, Switzerland; 3 Central Laboratory, HFR-Fribourg, 1700 Fribourg, Switzerland; 4 ADMed Laboratories, 2300 La Chaux-de-Fonds, Switzerland

**Keywords:** ferritin, matrix, analytical methods, population, iron deficiency, reference intervals

## Abstract

Objectives. To determine the ferritin inter-assay differences between three “Conformité Européenne” (CE) marked tests, the impact on reference intervals (RI), and the proportion of individuals with iron deficiency (ID), we used plasma and serum from healthy blood donors (HBD) recruited in three different Switzerland regions. Design and Methods. Heparinized plasma and serum from HBD were obtained from three different transfusion centers in Switzerland (Fribourg, Geneva, and Neuchatel). One hundred forty samples were recruited per center and per matrix, with a gender ratio of 50%, for a total of 420 HBD samples available per matrix. On both matrices, ferritin concentrations were quantified by three different laboratories using electrochemiluminescence (ECL), latex immunoturbidimetric assay (LIA), and luminescent oxygen channeling immunoassay (LOCI) assays, respectively. The degree of agreement between matrices and between the three sites/methods was assessed by Passing–Bablok and we evaluated the proportion of individuals deemed to have ID per method. Results. Overall, no difference between serum and heparinized plasma ferritin values was observed according to Passing–Bablok analyses (proportional bias range: 1.0–3.0%; maximum constant bias: 1.84 µg/L). Significant median ferritin differences (*p* < 0.001 according to Kruskal–Wallis test) were observed between the three methods (i.e., 83.6 µg/L, 103.5 µg/L, and 62.1 µg/L for ECL, LIA, and LOCI in heparinized plasma, respectively), with proportional bias varying significantly between ±16% and ±32% on serum and from ±14% to ±35% on plasma with no sign of gender-related differences. Affecting the lower end of RI, the proportion of ID per method substantially varied between 4.76% (20/420) for ECL, 2.86% (12/420) for LIA, and 9.05% (38/420) for LOCI. Conclusions. Serum and heparinized plasma are exchangeable for ferritin assessment. However, the order of magnitude of ferritin differences across methods and HBD recruitment sites could lead to diagnostic errors if uniform RI were considered. Challenging the recently proposed use of uniform ferritin thresholds, our results highlight the importance of method- and region-specific RI for ferritin due to insufficient inter-assay harmonization. Failing to do so significantly impacts ID diagnosis.

## 1. Introduction

Iron is an essential mineral required for various physiological and cellular processes in humans. It plays a major role in oxygen transport on the hemoglobin of erythrocytes but is also present in smaller amounts in all body cells as an essential component of ferroproteins or enzymes. Intracellular storage of unused iron is provided by ferritin to prevent damage associated with reactive oxygen species. Ferroportin is the ubiquitous transporter that mediates iron efflux from the cells. Erythropoiesis alone requires 1 mg of iron per 1 mL of erythrocytes, which means that approximately 20 mg of iron is used per day for hemoglobin production [1]. Most of the iron required for erythropoiesis is recycled by macrophages of the reticuloendothelial system, and only 0.1% of body iron is lost daily [2]. The recommended daily oral intake for adults aged 19–50 years of age is estimated to be 8 mg for men and 18 mg for women [3]. Regulation of iron metabolism is mediated by two hormones: hepcidin and erythroferrone [2]. Hepcidin can reduce iron absorption by inducing ferroportin endocytosis on the basolateral surface of enterocytes. By the same mechanism, it favors iron sequestration in macrophages. During inflammation, the expression of hepcidin is increased by cytokines [2]. Consequently, hepcidin induces a functional iron deficiency characterized by high serum ferritin levels. Erythroferrone suppresses hepcidin expression when iron is required for erythropoiesis.

It has been proven that total body iron can vary between individuals, especially depending on dietary habits, menstruation in women, genetic factors, or even ethnicity [4,5]. In 2016, iron deficiency anemia (IDA) was estimated to affect more than 1.2 billion people worldwide. Iron deficiency without anemia (IDWA) is also a common situation, affecting about twice as many patients [6]. These situations are major global problems, especially for women of reproductive age in low-income countries. To address this burden, the WHO launched an international action plan in 2014 to reduce anemia in women of reproductive age by 50% by 2025 [7]. In a proper nutritional context, iron deficiency is also an indicator of chronic blood loss; for example, it is the first sign of a malignant tumor of digestive origin.

Randomized controlled trials have shown that oral iron supplementation in IDWA with ferritin levels below 50 µg/L is effective in eliminating unexplained fatigue [8,9]. Iron supplementation in heart failure patients with ferritin levels <100 µg/L (or even higher <300 µg/L if transferrin saturation is below 20%) improved patient outcomes [10]. Some observational studies have shown an association between iron deficiency (ID) and restless legs syndrome, and experts recommend iron therapy when ferritin levels are <75 µg/L [11]. Finally, studies of iron therapy have shown improvements in cognitive and aerobic performance [12,13]. 

Although bone marrow aspirate is the gold standard for determining an individual’s total body iron stores, this invasive procedure has been gradually replaced in recent decades by measurement of serum or plasma ferritin, the extracellular portion of ferritin. In fact, serum ferritin is considered the most accurate biomarker for the diagnosis of iron deficiency [14]. However, little is known about its release in plasma and its contribution to iron homeostasis [6]. Ferritin is a protein complex composed of 24 subunits of heavy and light chains, which can store about 4500 iron atoms. This nanocage is present in all the body’s cells, but only in small fractions in the extracellular compartment [1].

Researchers have shown in mice that extracellular ferritin is likely to be released from macrophages via a non-classical secretory pathway rather than from damaged cells [15]. Recently, novel pathways such as ferritinophagy and ferroptosis have been discovered, opening perspectives for a better understanding and treatment of iron-related diseases as well as infections or cancer [16]. Finally, there has recently been an increased interest in ferritin-based vaccines, mainly due to their good immunogenicity and safety [17].

Numerous immunological methods are currently available for the measurement of ferritin, including non-radiometric assays (e.g., enzyme-linked immunoassay, enzyme-linked immunosorbent assay, or chemiluminescence), radiometric assays, and agglutination assays (e.g., turbidimetry or nephelometry) [18]. These methods have been adapted for use on automated systems, allowing rapid determination of this biological parameter.

However, it appears that ferritin values close to diagnostic or therapeutic decision thresholds are subject to variations between different methods. These variations are due to incomplete assay standardization, despite the introduction of several international ferritin reference standards since 1985 [19,20,21,22]. ID is prevalent worldwide and causes treatable health problems. Therefore, control of ferritin dosage is essential to ensuring quality medical care. Despite these limitations, the International Consortium for Harmonization of Clinical Laboratory Results recently stated that uniform ferritin thresholds should be used for IDA because there is sufficient inter-assay ferritin harmonization to allow medical decision-making [23].

In this study, we aimed to determine the inter-assay ferritin differences between three “Conformité Européenne” (CE)-labeled assays and their impact on the determination of reference intervals and the respective proportions of individuals with ID using plasma and serum matrices from first-time healthy blood donors (HBD) recruited in three different regional blood centers in Switzerland.

## 2. Materials and Methods

### 2.1. Design 

**Figure 1:**
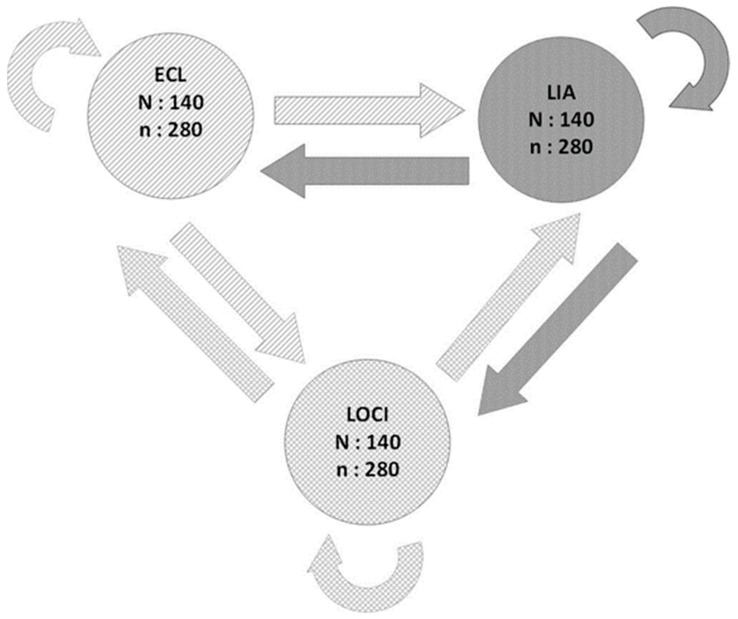
Flowchart showing the distribution of the 840 samples between laboratories. *N*: number of blood donors. *n*: number of samples. ECL: electrochemiluminescence. LIA: latex immunoturbidimetric assay. LOCI: luminescent oxygen channeling immunoassay.

Three blood centers were included in this study: Fribourg, Geneva, and Neuchâtel. Blood samples were collected from 70 men and 70 women at each blood center. Serum and heparinized plasma samples were collected simultaneously. The following data were collected: age at the time of sampling and sex. Each center had its own testing laboratory. All the samples were centrifuged less than 12 h after collection, aliquoted less than one hour after centrifugation, and frozen at −80 °C less than 8 h thereafter. Consequently, all samples were frozen at −80 °C no later than 21 h after collection. A visual check for hemolysis was systematically carried out before freezing. Each sample was aliquoted into three subsamples for distribution among the three laboratories (Figure 1). Transport between the centers was carried out at a temperature of −20 °C. Samples from each center were always transported together. 

### 2.2. Eligibility

This study included 420 blood donors from three different centers in Switzerland: Fribourg, Geneva, and Neuchâtel. To avoid the effect of blood donation on the ferritin level, only first-time blood donors and those who had not donated for at least one year before were selected. Knowing that a blood donation is equivalent to 250 mg of iron and that insensible iron losses are estimated at 1–2 mg per day, it takes a minimum of 250 days to compensate for the loss linked to a donation. A precautionary margin of 100 days avoids the bias associated with donors who have given several times a year [24]. All these donors were healthy adults, between 18 and 65 years old, and selected according to our national blood donor regulations [25].

### 2.3. Biological Measurement

Each routine laboratory had a different analytical method and performed the analysis on both matrices (serum and heparinized plasma) within one hour after thawing. The used methods were electrochemiluminescence (ECL) on a Cobas Pro (Hitachi High-Technologies Corporation for Roche, Tokyo, Japan) at the Fribourg laboratory, latex immunoturbidimetric assay (LIA) on a Cobas 8000 module c502 (Hitachi High-Technologies Corporation for Roche, Tokyo, Japan) at the Geneva laboratory, and luminescent oxygen channeling immunoassay (LOCI) on a Dimension Vista 1500 (Siemens Healthcare Diagnostics Inc., Newark, DE, USA) at the Neuchâtel laboratory. All routine laboratories participated regularly in external quality controls according to our national requirements, and their quality internal controls showed a coefficient of variation below 5% for normal values.

### 2.4. Statistical Analyses

The primary outcome was to compare ferritin values between matrices and analytical methods and how these would impact the lower end of the respective reference interval (RI). The respective reference intervals were commonly set at the 2.5 centile and 97.5 centile of the values obtained per method/site.

The second outcome was the proportion of patients considered iron deficient per method/site. According to the WHO guidelines, ID was defined as ferritin levels below 15 μg/L for our cohort [5].

The sample size was calculated using a comparison of expected medians with a difference of 10 µg/L (α = 0.05, power = 0.9), and the minimum sample size was estimated to be 84 per group. Statistical analyses were carried out using Analyse-it software version 5.80.2 to perform Passing–Bablok analyses to compare serum and heparinized plasma. These analyses were reported with 95% confidence intervals (95% CI).

Kruskal–Wallis and Mann–Whitney tests were used to compute differences between continuous variables, while the Chi-square (χ^2^) test was used to assess differences between proportions. Analyses were performed using Statistica software version 13.5. Significance was considered at the *p* < 0.05 level.

## 3. Results

### 3.1. Samples and Subjects

All samples were collected between 7 June and 26 July 2021. Sample analysis was performed by each laboratory between 8 June 2021, and 10 February 2022. The mean freezing time was 166 days for serum and heparinized plasma, ranging from a minimum of 0 days to a maximum of 246 days. The preanalytical procedure was followed for all samples, and all of them were analyzed.

The mean age by center was 38.6 [range: 18.3–62.9], 39.9 [18.0–64.3], and 43.4 [18.3–66.6] years old for Neuchâtel, Fribourg, and Geneva centers, respectively.

A total of 2520 ferritin assays were performed, including 1260 on the serum matrix and 1260 on the heparinized plasma matrix. The median ferritin for all the samples analyzed by all the laboratories was 78.1 µg/L (range: 2.4–1188.0 µg/L) and 80.1 µg/L (range: 2.0–1162.0 µg/L) for serum and heparinized plasma matrix, respectively. By separating genders and matrices, for men (*N* = 630), the median values were 123.3 µg/L (range: 5.1–1188.0 µg/L) for serum and 124.0 (range: 4.5–1162.0 µg/L) for heparinized plasma; and for women (*N* = 630), the median values were 52.2 µg/L (range: 2.4–302.0 µg/L) for serum and 52.8 (range: 2.0–291.0 µg/L) for heparinized plasma.

### 3.2. Comparison of Matrices

**Figure 2:**
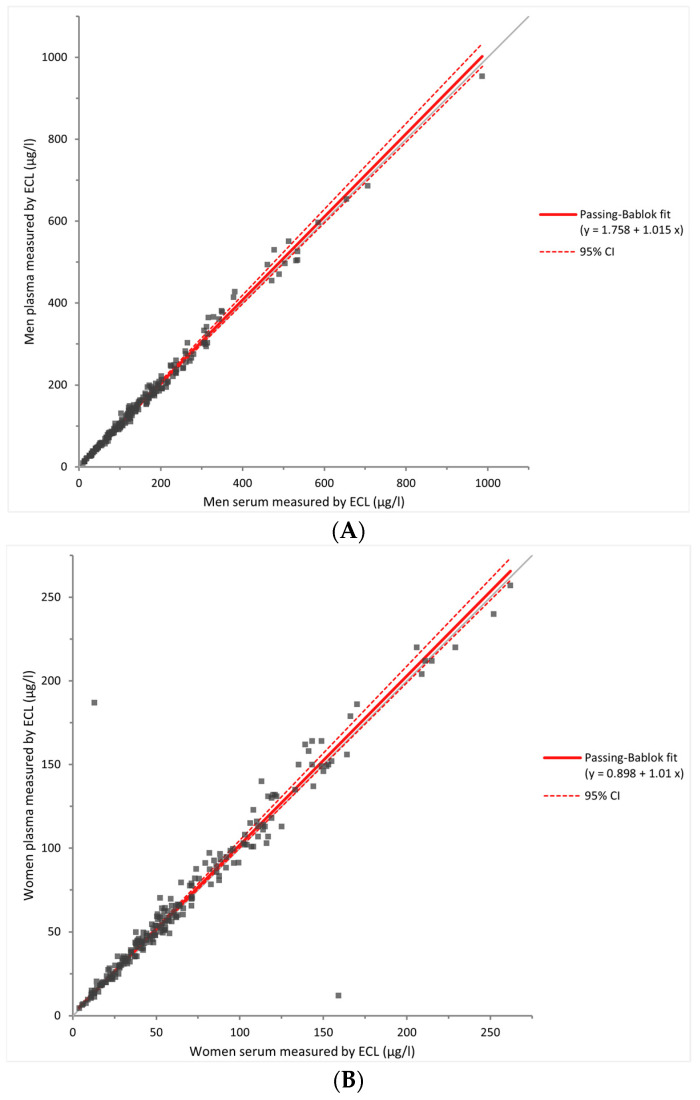

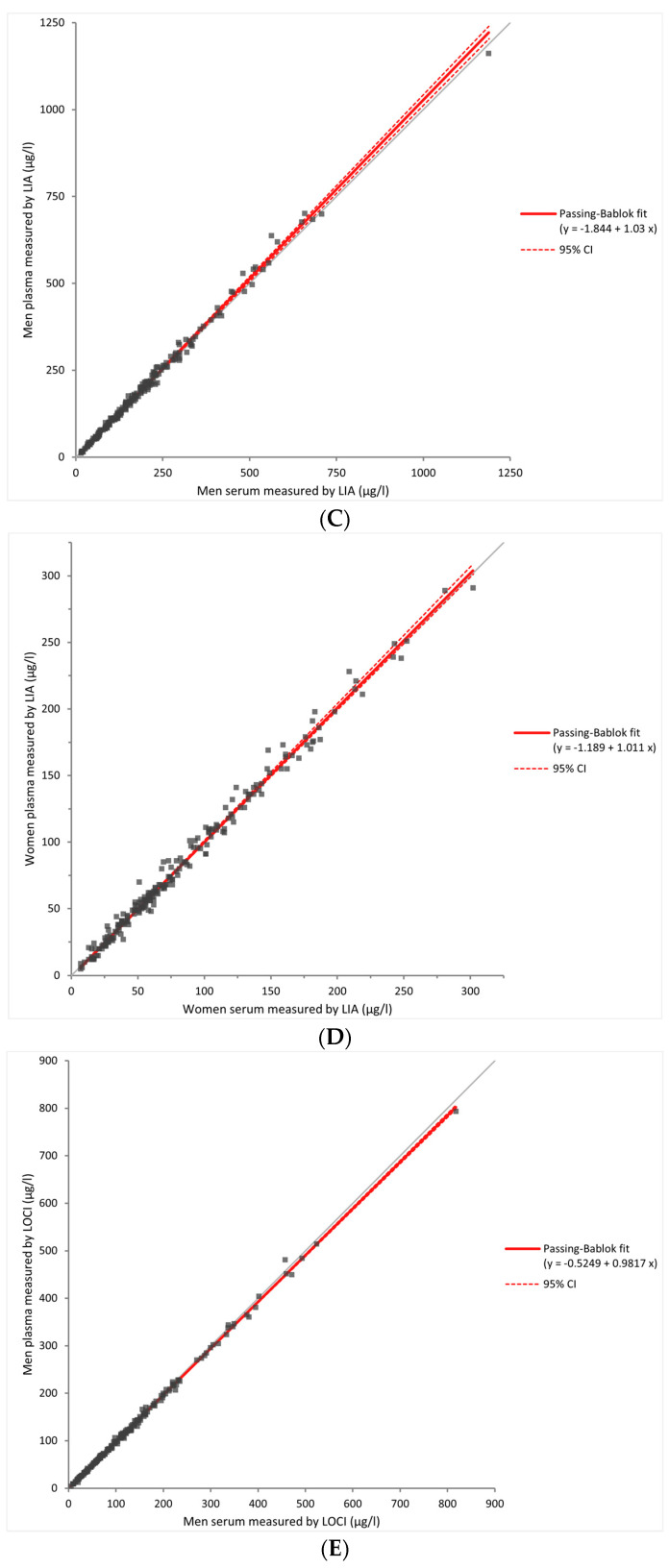

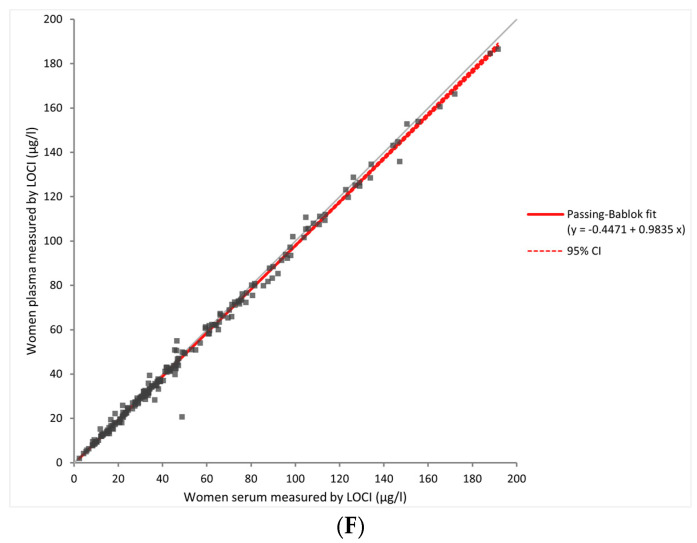
Comparison of matrices between serum and heparinized plasma. Passing–Bablok analyses were performed by ECL (**A**,**B**), LIA (**C**,**D**), and LOCI (**E**,**F**) for men and women, respectively. ECL: electrochemiluminescence. LIA: latex immunoturbidimetric assay. LOCI: luminescent oxygen channeling immunoassay.

Ferritin in serum and heparinized plasma values were compared with each method using the Passing–Bablok test, separating men (*N* = 210) and women (*N* = 210). If the values of ferritin were not included in the 95% CI, the two methods were considered significantly different (Figure 2). To determine whether there was agreement or not between these two matrices, we analyzed the slope of the curve, which should be equal to 1 if the values were identical. Using the ECL method, Passing–Bablok analyses demonstrated a slope of the curve of 1.015 [IC 95%: 0.988–1.047] for men (i.e., proportional bias of 1.5%) and 1.010 [IC 95%: 0.987–1.042] for women (i.e., proportional bias of 1.0%) (Figure 2A,B). Using the LIA method, we observed a slope of the curve of 1.030 [IC 95%: 1.014–1.047] for men (i.e., proportional bias of 3.0%) and 1.011 [IC 95%: 1.000–1.029] for women (i.e., proportional bias of 1.1%) (Figure 2C,D). Using the LOCI method, we observed a slope of the curve of 0.982 [IC 95%: 0.977–0.987] for men (i.e., proportional bias of 1.8%) and 0.984 [IC 95%: 0.976–0.991] for women (i.e., proportional bias of 1.6%) (Figure 2E,F). In all of these analyses, the constant bias observed ranges from a minimum of 0.45 µg/L to a maximum of 1.84 µg/L.

### 3.3. Method Comparison

**Figure 3:**
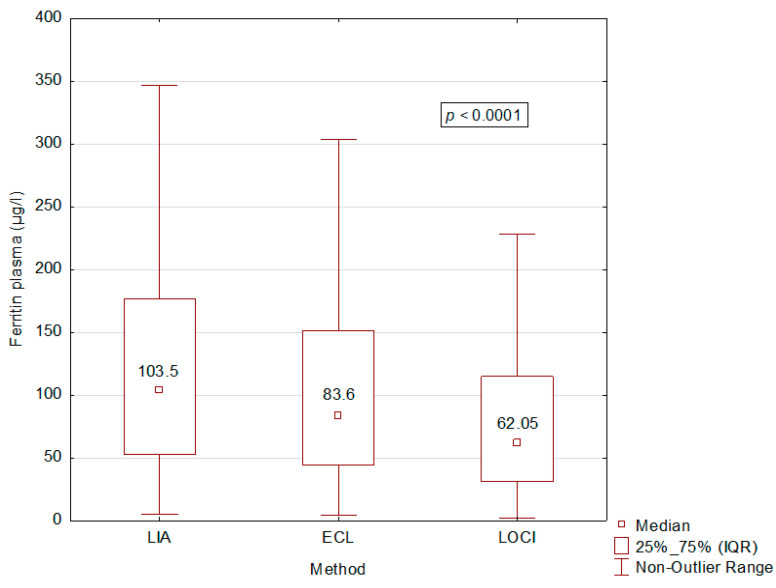
Comparison of the three methods using the Kruskal–Wallis test. ECL: Electrochemiluminescence. LIA: latex immunoturbidimetric assay. LOCI: luminescent oxygen channeling immunoassay.

**Figure 4:**
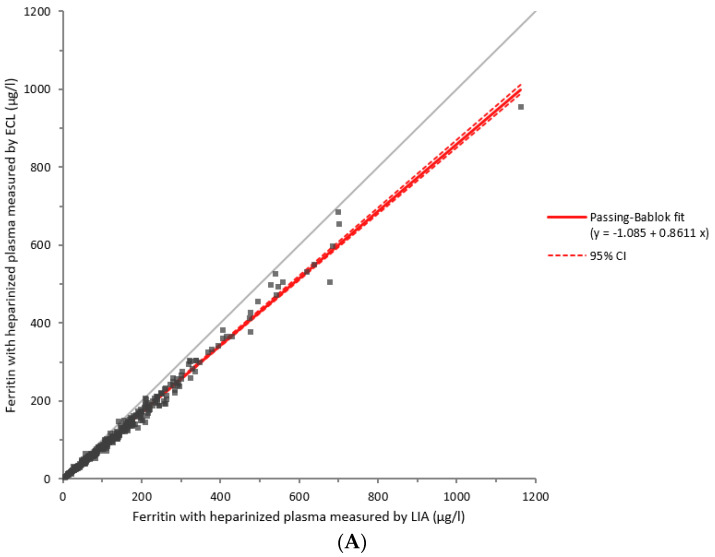

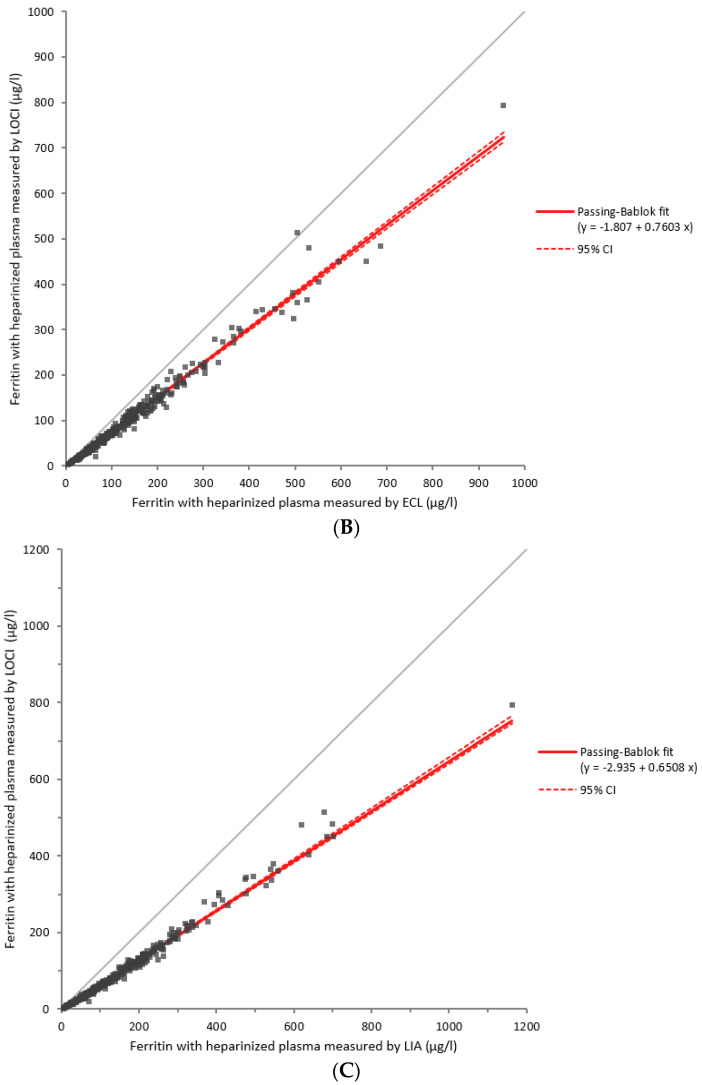
Comparison of the three methods two by two using the Passing–Bablok analyses, performed with heparinized plasma between the ECL and LIA methods (**A**), LOCI and ECL methods (**B**), and LOCI and LIA methods (**C**). ECL: electrochemiluminescence. LIA: latex immunoturbidimetric assay. LOCI: luminescent oxygen channeling immunoassay.

Ferritin measurements were performed for all samples (*n* = 840) in each of the three laboratories to compare these different analytical assays. Ferritin medians with heparinized plasma matrix were 83.6 µg/L [IC 95%: 72.6–94.7 µg/L] measured by the ECL assay, 103.5 µg/L [IC 95%: 90.9–116.1 µg/L] by the LIA assay, and 62.1 µg/L [IC 95%: 53.4–70.7 µg/L] by the LOCI assay (Table 1). Using the Kruskal–Wallis test, we showed a significant difference between these three assays with a value of *p* ≤ 0.0001 for serum and heparinized plasma matrices (Figure 3). Using the Mann–Whitney test, comparisons of the assays two by two were also all significant, whatever the matrix considered. With a serum matrix, *p*-values were *p* = 0.000973 for a comparison between ECL and LIA, *p* = 0.000103 for a comparison between ECL and LOCI, and *p* ≤ 0.000001 for a comparison between LIA and LOCI. With a heparinized plasma matrix, *p*-values were *p* = 0.004540 for a comparison between ECL and LIA, *p* ≤ 0.000001 for a comparison between ECL and LOCI, and *p* ≤ 0.000001 for a comparison between LIA and LOCI. Passing–Bablok analyses confirm these significant differences (Figure 4). Furthermore, the three analytical methods in our study showed good measurement accuracy.

### 3.4. Impact on Reference Intervals

The on-site reference intervals are presented in Table 2. At the 2.5th percentile, the ferritin values with heparinized plasma matrix were 15.79 µg/L (for men) and 10.66 µg/L (for women) for the ECL method, 18.35 µg/L (for men) and 12.00 µg/L (for women) for the LIA method, and 10.03 µg/L (for men) and 7.75 µg/L (for women) for the LOCI method. The same percentile values with serum matrix were 14.88 µg/L (for men) and 10.69 µg/L (for women) for the ECL method, 21.68 µg/L (for men) and 13.45 µg/L (for women) for the LIA method, and 11.50 µg/L (for men) and 8.32 µg/L (for women) for the LOCI method.

### 3.5. Impact on the Proportion of Individuals with ID According to Method

Using the ID cut-off of 15 μg/L, the proportions of ID per method were 4.76% (20/420) for the ECL method, 2.86% (12/420) for the LIA method, and 9.05% (38/420) for the LOCI method. These differences were non-significant between ECL and LIA (*p* = 0.149). However, these differences were significant between LOCI and LIA (*p* ≤ 0.0001) and between LOCI and ECL (*p* = 0.01). These values were determined with a heparinized plasma matrix.

### 3.6. Impact on the Proportion of Individuals with ID According to Site

Using the ID cut-off of 15 μg/L for ferritin measurements performed by the ECL assay, the proportions of ID per site were 0.71% (1/140) for Fribourg, 10.71% (15/140) for Geneva, and 2.86% (4/140) for Neuchâtel. These values were determined with a heparinized plasma matrix.

### 3.7. Method Comparison near the Low Clinical Decision Threshold

We also carried out nested analyses focusing on ferritin values lower than 100 µg/L (*n* = 241), close to the clinical decision thresholds. Using the Kruskal–Wallis test, we showed a significant difference between these three assays (*p* ≤ 0.0001). Using the Mann–Whitney test, comparisons of the methods two by two were also always significant: *p*-values were *p* = 0.000341 for the comparison between ECL and LIA, *p* ≤ 0.000001 for the comparison between ECL and LOCI, and *p* ≤ 0.000001 for the comparison between LIA and LOCI. These values were determined with a heparinized plasma matrix.

### 3.8. Population Comparison

To compare populations between the three centers, we used the Kruskal–Wallis test with the two matrices and the three methods. Results showed a significant difference in all six situations, with a value of *p* ≤ 0.0001. However, using the Mann–Whitney test, we found conflicting results (Supplementary Materials).

## 4. Discussion

In this prospective study, we simultaneously evaluated the effect of two matrices (serum and heparinized plasma) and three analytical methods (ECL, LIA, and LOCI) on the measurement of human ferritin, performing 2520 ferritin assays. We found excellent agreement between serum and heparinized plasma ferritin values, suggesting commutability between these two matrices for each of these three analytical methods. Although the ferritin assay is based on an immunological interaction, serum is not the only matrix that can be used. The small but significant differences between serum and heparinized plasma matrices, with a maximum proportional bias of 3.0% and a maximum constant bias of 1.84 µg/L, would be within the 6.99% measurement uncertainty recently determined by Rovegno et al. in their 2023 study [26]. The present data confirm previous studies, such as those of Birgegård in 1980, which compared samples from nine subjects, or more recently, those of Snozek et al. in 2021, which compared serum and heparinized plasma, in which a correlation of 0.9992 was retrieved between these two matrices [27,28]. In their 2023 study, Majoni et al. observed identical results using the chemiluminescent microparticle immunoassay (CMIA) method, but an overestimation of 5% with serum compared to heparinized plasma matrix using a two-phase immunometric method [29].

This commutativity means that, for each clinical situation, the most rational sampling strategy can be chosen, saving both patient blood volume and preanalytical procedures. It is also worth pointing out that a sample taken with an anti-coagulant, especially heparin, then centrifuged yields a greater volume of plasma for analysis than a serum sample. Finally, plasma samples are not affected by the in vitro coagulation process [30].

The key finding of our study is that substantial and significant differences in ferritin values were observed among the three CE-marked methods evaluated.

The median differences in ferritin values in the entire cohort ranged from 17.0 µg/L to 37.2 µg/L and from 19.9 µg/L to 41.5 µg/L for serum and heparinized plasma matrix, respectively. The differences observed in our study also substantially affected the lower end of the respective reference intervals, with differences up to 10.2 µg/L depending on the method performed. Accordingly, the proportions of a ferritin-based ID diagnosis per method vary from 2.86% to 9.05% in our study.

These inter-method differences are consistent with those shown by Choy et al. in 2022, who compared 19 samples with different ferritin levels using five different analytical methods [21]. On the other hand, in their 2018 systematic review and meta-analysis, which included 187 studies, Garcia-Casal et al. showed a comparable correlation for this biomarker between several common analytical methods [18]. Indeed, an overall correlation coefficient of 0.981 was observed when comparing non-radiometric and radiometric methods with each other, as well as when comparing the subtypes of methods included in these categories.

Our study, which analyzed samples from 420 subjects simultaneously under standardized preanalytical conditions, confirms the inter-method variability for common clinical values. This inevitably leads to a risk of diagnostic error and inadequate patient care [8,9,10,11,12,13]. This difference also seriously hampers the evaluation of the therapeutic efficacy of oral iron after a trial treatment of a few weeks. These methodological differences could be explained by the immunological issues involved in test manufacturing, which implies that each laboratory should define its own reference intervals.

This means that it is important for the clinical management of a patient that the analyses are carried out in the same laboratory. A similar conclusion was reached by researchers studying ferritin values among patients with chronic kidney disease [29]. Indeed, in this study, 179 blood samples were analyzed for ferritin using two different assays, with an observed bias of up to 49%. It is nevertheless important to specify that in this latter study, the median ferritin level was higher than in our study and is therefore further away from the clinical decision threshold of ID without kidney or heart failure.

It also seems crucial for clinicians managing iron deficiency to be aware of these methodological differences when assessing ID. In fact, as mentioned above, significant differences were also found when investigating only the lower values, close to the clinical decision point (values of ferritin <100 µg/L). According to the “WHO guideline on use of ferritin concentrations to assess iron status in individuals and populations”, critical ferritin values range from 12 µg/L to 70 µg/L depending on age, gender, and clinical status [5]. If a physician works with different laboratories, he or she runs the risk of therapeutic management errors.

In our study, we also suspected regional variations in ferritin concentrations, although differences were not always significant. As demonstrated in several previous studies, this can be explained in various ways, such as dietary habits, menstruations for women, genetic factors, or even ethnicity [4,5]. Moreover, there are variations in ferritin levels within the same population over time, as demonstrated in Australia between 1995 and 2005, with an increase of 21% in ferritin levels for men and 10% for women during this ten-year period [31]. The addition of population differences to differences between analytical methods could increase, in some situations, the risk of error in patient management.

Our study has several limitations. First, the duration of sample storage was quite variable among subjects in all laboratories, and we did not investigate the effect of storing frozen samples on analytical results over time. Second, information regarding dietary habits, genetic factors, ethnicity, or contraceptive treatment of women is lacking. This prevents us from defining reference intervals adapted to specific clinical contexts, such as a particular diet or comorbidity. Finally, our data are based on a healthy population of blood donors, which includes some subjects with iron deficiency but very few patients with hyperferritinemia. Our data are therefore far from those used to manage hemochromatosis. It would also be useful to study these analytical differences in a population of iron-deficient patients with renal diseases or inflammation [29].

## 5. Conclusions

While ferritin is still considered the most powerful marker for diagnosing iron deficiency, its measured values vary significantly depending on the analytical method used. This can be particularly critical when the values are close to the clinical decision threshold. However, our study shows that the three assays that we evaluated can be used independently with serum or heparinized plasma matrices.

## Figures and Tables

**Table 1:** Medians and 25th and 75th percentiles of ferritin assays with each method separating men (*n* = 210) and women (*n* = 210) with a heparinized plasma matrix.

	ECL		LIA		LOCI	
	Ferritin (µg/L)		Ferritin (µg/L)		Ferritin (µg/L)	
	Men	Women	Men	Women	Men	Women
Median	133.5	54.8	158.5	65.0	98.9	37.1
25th percentile	76.7	34.1	92.5	40.3	55.6	22.5
75th percentile	203.3	94.4	244.8	110.0	156.3	70.6

ECL: electrochemiluminescence. LIA: latex immunoturbidimetric assay. LOCI: luminescent Oxygen channeling immunoassay.

**Table 2:** Reference interval (2.5th and 97.5th percentile) values for the three methods, for each matrix, and for men (*N* = 210) and women (*N* = 210). ECL: electrochemiluminescence. LIA: latex immunoturbidimetric assay. LOCI: luminescent oxygen channeling immunoassay. 2.5p: 2.5th percentile. 97.5p: 97.5th percentile.

		Plasma		Serum	
		2.5p (µg/L)	97.5p (µg/L)	2.5p (µg/L)	97.5p (µg/L)
ECL	Men	15.79	529.10	14.88	533.33
	Women	10.66	210.20	10.69	208.33
LIA	Men	18.35	632.95	21.68	575.40
	Women	12.00	235.75	13.45	236.83
LOCI	Men	10.03	439.62	11.50	444.72
	Women	7.75	150.98	8.32	149.74

## Data Availability

All data can be obtained by email request from the corresponding author.

## Supplementary Materials

The following supporting information can be downloaded at: https://www.mdpi.com/article/10.3390/diagnostics14040386/s1, Supplementary Materials S1. Details of the population comparison using both matrices and the three methods. The population of each center was 140 subjects. * significant difference with *p* < 0.05. ECL: electrochemiluminescence. LIA: latex immunoturbidimetric assay. LOCI: luminescent oxygen channeling immunoassay.
